# Supplementary material for: The causal relationship between the human gut microbiota and pyogenic arthritis: a Mendelian randomization study
Source: Front Cell Infect Microbiol. 2024 Nov 26;14:1452480. doi: 10.3389/fcimb.2024.1452480 (PMC11629706; doi:10.3389/fcimb.2024.1452480)
Supplement: Supplementary file 1 [file DataSheet1.zip › mendelian test/ebi-a-GCST90027584.csv_leave_one_out.pdf]

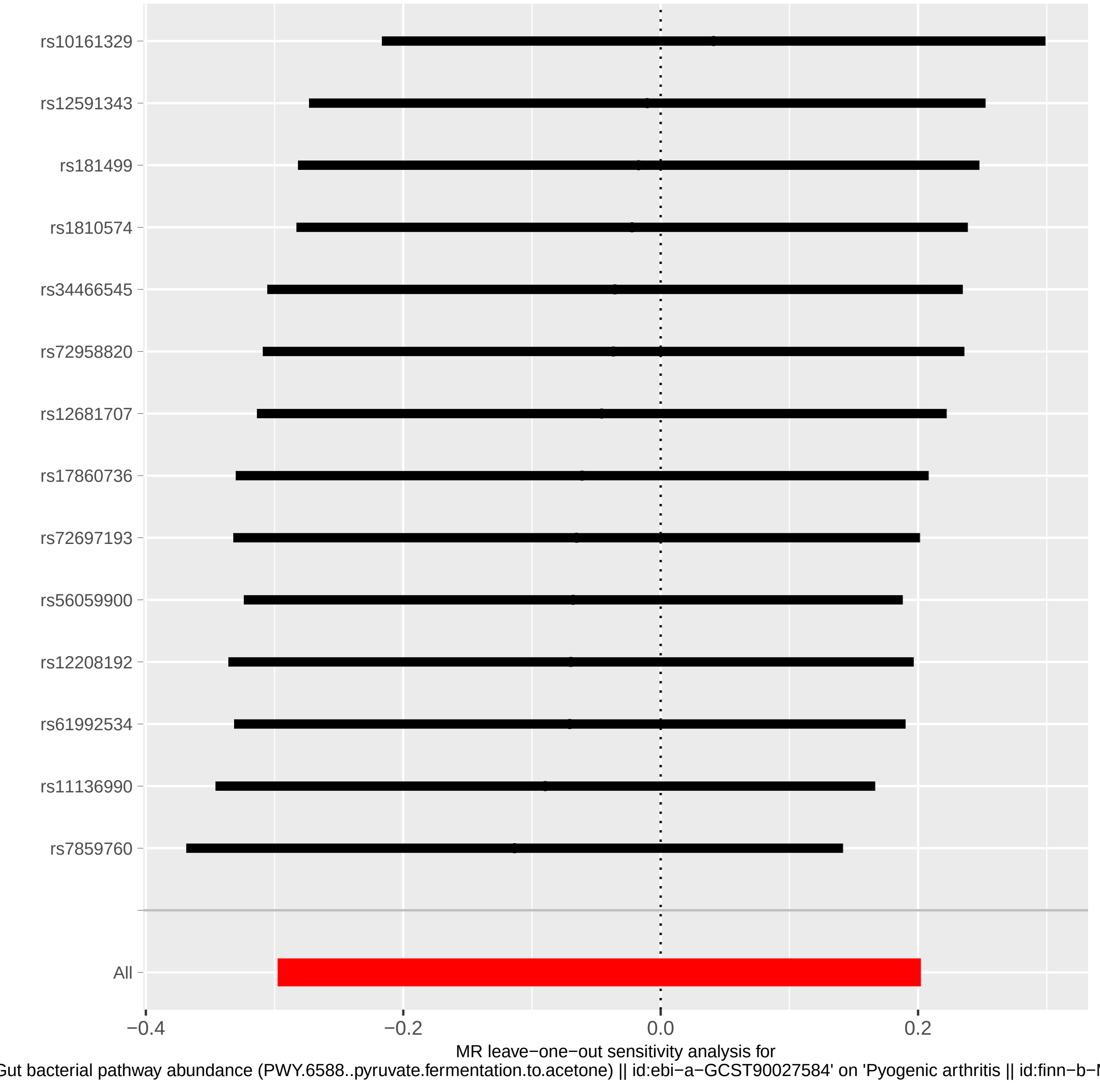
rs10161329
rs12591343
rs181499
rs1810574
rs34466545
rs72958820
rs12681707
rs17860736
rs72697193
rs56059900
rs12208192
rs61992534
rs11136990
rs7859760
All
−0.4
−0.2
0.0
0.2
MR leave−one−out sensitivity analysis for
Gut bacterial pathway abundance (PWY.6588..pyruvate.fermentation.to.acetone) || id:ebi−a−GCST90027584' on 'Pyogenic arthritis || id:finn−b−M
